# Characterizing Social Functioning in School-Age Children with Sensory Processing Abnormalities

**DOI:** 10.1007/s10803-021-05050-4

**Published:** 2021-05-06

**Authors:** T. St. John, A. Estes, K. K. Begay, J. Munson, M. A. Reiter, S. R. Dager, N. Kleinhans

**Affiliations:** 1 Department of Speech and Hearing Sciences, University of Washington, Seattle, WA USA; 2 University of Washington Autism Center, University of Washington, Seattle, WA USA; 3 Center On Human Development and Disability, University of Washington, Seattle, WA USA; 4 School of Education, University of Washington, Tacoma, WA USA; 5 Department of Psychiatry and Behavioral Sciences, University of Washington, Seattle, USA; 6 San Diego State University/ UC San Diego Joint Doctoral Program in Clinical Psychology, San Diego, CA USA; 7 Department of Radiology, University of Washington School of Medicine, Seattle, WA USA

**Keywords:** Sensory abnormalities, Social functioning, Autism spectrum disorder

## Abstract

Children with sensory abnormalities (SAs) have a variety of social problems resulting in poorer social functioning than children with typical development (TD). We describe the relationship between SAs and social functioning in school-age children with SAs, children with TD and a clinical comparison sample of children with autism spectrum disorder (ASD). Children with SAs demonstrated impaired social functioning on standardized measures. Children with SAs demonstrated worse social functioning than children with TD and equivalent social functioning to children with ASD. Increased SAs were associated with poorer social functioning across all groups. The results suggest that children with SAs experience clinically significant problems with social functioning and future research is needed to develop interventions to support social functioning in this population.

## Introduction

Sensory processing involves a cascade of events including the registration, organization, and interpretation of incoming information from the senses (Suarez, 2012). Inability to properly process visual, auditory, tactile, olfactory, gustatory, and proprioceptive input from the environment leads to sensory hyper- or hypo-sensitivity, and to difficulties modulating behavior (Koziol et al., 2011). Importantly, hypo- and hyper-sensory-sensitivity can occur simultaneously in an individual and may be present across multiple sensory domains (Baranek, 2002). Sensory abnormalities (SAs) are surprisingly common among school-aged children. A recent epidemiological study estimated that 8% of school-aged children experience SAs (Jussila et al., 2020), with higher rates reported in some studies (16.5%; Ben-Sasson et al., 2009). SAs affect 35%–95% of children with developmental disorders, including autism spectrum disorder (ASD) (Rogers & Ozonoff, 2005; Allen & Casey, 2017). SAs can affect daily functioning, learning, and social skills (Bar-Shalita et al., 2008; Koenig & Rudney, 2010; Elbasan et al., 2012; Dunn, 2014; Miller et al., 2017) and may account for some of the heterogeneity in social functioning among school-age children but further research is needed.

Social functioning (an individual’s behavior in a social environment, social skills and interactions with others; Green, 1996; Yager & Ehmann, 2006; Beauchamp & Anderson, 2010), is critical during school-age because of its association with academic achievement (Malecki & Elliott, 2002; Walker & Nabuzoka, 2007) and emotional well-being (Nangle et al., 2003; Lodder et al., 2017). Children with SAs demonstrate problems with social functioning such as poor social problem-solving, reduced empathic concern, and difficulty reading social cues (Ben-Sasson et al., 2009; Cosbey et al., 2012). Greater challenges interacting with peers during play than children with TD have also been reported (Cosbey et al., 2012; Armstrong et al., 2013). Children with SAs were shown to differ from TD peers on a number of dimensions related to social functioning during recess (Cosbey et al., 2012). Children with SAs engaged in significantly less social play and experienced more frequent and prolonged conflict with peers during play than children with TD. Children with SAs also made fewer relationship repair attempts (e.g., apologizing), were sought out less frequently for play, and demonstrated more difficulty responding to social cues (e.g., responding to cues of boredom, annoyance, or disinterest) than TD peers. Although this study was small (12 per group), the observational data provides some of the first direct evidence of social functioning deficits in children with SAs in a naturalistic environment with peers.

The relationship between SAs and social functioning has been studied more extensively in children with ASD. SAs across multiple sensory modalities (vision, hearing, taste, touch) are associated with a range of social functioning difficulties including reading social cues, responding to other’s social bids, and understanding the emotions of others (see Thye et al., 2018 for review). For example, greater atypical visual exploration in children with ASD was found to be associated with lower scores on measures of social skills (Hellendoorn et al., 2014). Increased social impairments on the Autism Diagnostic Observation Schedule (ADOS) and Autism Diagnostic Interview, Revised (ADI-R) were related to increased tactile hypo-responsiveness and tactile sensory seeking behaviors in children with ASD (Foss-Feig et al., 2012). Additionally, atypical processing of emotion in voices (e.g., discriminating happy versus angry) may impact processing of socially relevant auditory information (Fan & Cheng, 2014). Finally, worse olfactory identification has been associated with greater difficulties maintaining conversations (Bennetto et al., 2007). However, even in this population, significant gaps in the literature exist. A majority of the evidence comes from laboratory-based studies, using experimental measures of sensory processing, limiting the generalizability of the results. Parent report has also demonstrated associations between SAs and social functioning difficulties in children with ASD, providing convergent evidence for laboratory-based findings (Liss et al., 2006; Hilton et al., 2007; Baker et al., 2008).

Most research has focused on circumscribed domains of social functioning (i.e., adaptive functioning or reciprocal social behavior or peer relationships). However, social functioning is multi-faceted and a more comprehensive approach to understanding the relationship between SAs and social functioning is needed (Gree-Walker et al., 1994; Beauchamp & Anderson, 2010). In addition, few studies have included measures of communication despite the fact that communication deficits explain variability in social functioning among children with developmental disabilities (Venter et al., 1992; Leonard et al., 2011; Park et al., 2012; Staikova et al., 2013). For example, in sample of 28 children with ADHD, language skills mediated the relationship between ADHD symptoms and social skills (Staikova et al., 2013). Similarly, associations between communication and social skills have been found in children with ASD (Venter et al. 1992). Accounting for differences in communication skills could allow for better assessment of heterogeneity in social functioning.

The purpose of the present study was to (1) evaluate social functioning in children with SAs without developmental disabilities (2) contrast social functioning in children with SAs with two well-characterized, age-matched clinical comparison groups; children with ASD and TD, and (3) determine the relationship between SAs and social functioning across the full sample of children (SAs, ASD, TD) to better capture heterogeneity in SAs across clinical and non-clinical conditions.

## Method

Participants were 135 children between the ages of 8–13 with SAs (n = 44), ASD (n = 43), and TD (n = 48) who participated in a larger study focused on understanding the biochemical, brain, and behavioral correlates of sensory sensitivity in school-age children (Social and Sensory Processing Study, see Sweigert et al., 2020). Participant characteristics are presented in Table 1. Participants were recruited from the Seattle metropolitan area via flyers, online recruitment postings, and a research registry at the University of Washington. Participants were also recruited via medical records screening. Medical records were screened using key terms (e.g., sensory sensitivity) and prospective participants were sent a recruitment letter. All study procedures were approved by the University of Washington Human Subjects Division Institutional Review Board. Written informed consent was obtained from each participant’s parent and verbal assent was obtain from each participant.

**Table 1:** Participant characteristics

	SAs^a^		ASD^b^		TD^c^			
	M	SD	M	SD	M	SD	*F*	*p*-value
Sex (Male:Female)	38:6		39:4		41:7		χ^2^ = 0.64	.726
Race (White:Non-White)	34:10		36:7		37:11		χ^2^ = 0.76	.682
Age	10.03	1.63	10.28	1.64	10.32	1.44	0.48	.622
WASI FSIQ	115.64	18.12	112.33	17.9	118.79	11.7	1.84	.163
WASI PIQ	113.02	16.8	114.91	14.33	115.06	13.02	0.265	.768
WASI VIQ	114.75	18.07	107.33	21.21	118.33	12.33	4.46	.011
Vineland-II ABC^d^	85.35	11.72	80.15	10.81	104.83	12.41	54.64	< .001
Vineland-Communication ^d^	88.19	10.98	83.95	11.41	105.77	11.56	47.15	< .001
Expressive (v-scale score)	12.77	1.9	11.29	1.7	15.77	2.5	53.92	< .001
Receptive (v-scale score)	11.95	2.53	11.37	2.22	15.75	1.85	53.70	< .001
Written (v-scale score)	14.30	3.06	13.83	3.28	15.83	2.24	5.95	.003
ADOS-2 CSS Total^e^	3.16	2.94	7.12	1.95	1.46	.82	123.14	< .001
ADOS-2 Social Affect CSS^f^	3.73	2.56	7.37	1.94	2.00	1.24	86.76	< .001
ADOS-2 RRB CSS^g^	3.50	2.26	6.67	2.33	1.77	1.77	61.85	< .001

	n	%	n	%	n	%		
K-SADS ADHD	26	59	21	49	0	0		
K-SADS Anxiety	12	27	11	26	0	0		
K-SADS Depression	7	16	0	0	0	0		
K-SADS ODD	5	11	3	7	0	0		

^a^n = 44

^b^n = 43

^c^n = 48

^d^SAs n = 40, ASD n = 40, TD = 47

^e^ASD > SA > TD

^f^ASD > SA > TD

^g^ASD > SA > TD

Initial telephone screening interviews were conducted with parents to determine study eligibility and group assignment. All participants were required to have an FSIQ above 70 and not meet criteria for intellectual disability. 151 participants (50 SAs, 51 ASD, 50 TYP) were enrolled in the study after initial screening and were invited for an in-person evaluation. The SAs group all scored at or above two standard deviations above the mean of the normative sample (“much more than others”) on one or more sensory processing domains of the Child Sensory Profile, Second Edition (CSP-2; Dunn, 2014). Children in the SAs group were excluded if they reported a history of ASD, first-degree relative with ASD, or a known inherited genetic disorder during the initial screening interview. Children in the ASD group each had a previous ASD diagnosis. Children with ASD were excluded if they had a history of schizophrenia or other psychotic disorder or a known inherited genetic disorder. One child initially in the SAs group was transferred to the ASD group after the research assessment established that they met DSM-5 criteria for ASD. Children in the TD group were excluded for prior diagnosis of ASD, ADHD, intellectual disability, or other psychiatric/developmental disorder, having a first-degree relative with ASD, and any score on the CSP-2 at or above two standard deviations above the mean (“much more than others”).

Group assignment was confirmed through in-person evaluations by a licensed clinical psychologist or psychology graduate student under the supervision of the study’s lead licensed clinical psychologist (TS). All children in the ASD group met clinical best estimate (CBE) diagnosis using the Autism Diagnostic Interview, Revised (ADI-R; Lord et al., 1994), Autism Diagnostic Observation Schedule, Second Edition (ADOS-2; Lord et al., 2012), and DSM-5 ASD criteria (American Psychiatric Association, 2013). Children in the SAs and TD groups did not meet criteria for CBE diagnosis for ASD. The Wechsler Abbreviated Scale of Intelligence (WASI; Wechsler, 1999) was used to assess cognition and intellectual performance. The Kiddie Schedule for Affective Disorders and Schizophrenia (K-SADS-PL DSM-5; Kaufman et al., 1997) was used to screen for associated psychiatric conditions, as described above, for the children with ASD and TD. Seventeen participants were disqualified after an in-person clinical evaluation (e.g., FSIQ < 70, etc.) resulting in a final sample of 135 participants (44 SPD, 43ASD, 48 TD).

Descriptive information on participants is provided in Table 1. There were no group differences in age, gender, or race. On the WASI there were no significant group differences for FSIQ or PIQ but the ASD group had significantly lower VIQ scores than the TD group (*p* = 0.01). On the K-SADS-PL DSM-5, nearly half of the sample of children with SAs demonstrated symptoms of ADHD, approximately one-third, symptoms of anxiety, a little less than a quarter, symptoms of depression, and over 10 percent reported symptoms of oppositional defiant disorder. Comparable rates of psychiatric symptoms were observed in the ASD group, with the exception of depressive symptoms. The TD group did not report any clinically significant symptoms on the K-SADS-PL DSM-5.

## Measures

### Sensory Symptoms

The Children’s Sensory Profile, Second Edition (CSP-2; Dunn, 2014) is a standardized, 86-item parent-report measure that assesses sensory behaviors in children ages 3 to 14 years. It is intended to capture sensory experiences occurring in daily life. Caregivers rate the frequency of their child’s response to various sensory experiences on a 5-point scale (1 = *almost never* to 5 = *almost always*). The CSP-2 yields four sensory processing pattern scores including Seeking/Seeker, the degree to which a child seeks out sensory experiences, Avoiding/Avoider, the degree to which a child is overwhelmed by sensory experiences, Sensitivity/Sensor, the degree to which a child detects sensory input, and Registration/Bystander, the degree to which a child misses sensory cues. Total raw scores are calculated for each domain and are associated with five classifications based on standard deviations from the mean of the normative sample (− 2 SDs below the mean = “Much Less Than Others”, − 1 to − 2 SD’s below the mean = “Less Than Others”, − 1 to + 1 SD below and above the mean = “Just Like the Majority of Others”, + 1 and + 2 SD’s above the mean = “More Than Others”, and + 2 SD’s above the mean = “Much More Than Others”). The CSP-2 has been validated in clinical populations such as ASD and ADHD. Test–retest ranges from 0.83—0.97 and inter-rater from 0.70 to 0.80 (Dunn, 2014). In the current study, the overall Cronbach’s alpha was 0.89 for the overall sample (ASD = 0.84 and SAs = 0.89).

### Social Functioning

The Social Responsiveness Scale–Second Edition (SRS-2) is a 65-item parent-report, rating scale that measures deficits in reciprocal social behavior and restricted and repetitive behaviors characteristic of children with ASD (Constantino & Gruber, 2012). Items on the SRS-2 measure the ability to recognize social cues, how one interprets other’s social behavior, reciprocal social communication during social interactions, and motivation to engage in social interactions as well as restricted and repetitive interests and behaviors. Although the SRS-2 is most commonly used to assesses ASD-related impairments, empirical evidence have shown good variation in individuals without ASD which suggests that it is a reasonable index of reciprocal social behavior (Coon et al., 2010; Ebstein et al., 2010; Wagner et al., 2019). Higher scores indicate more impairment in reciprocal social behavior. Unusually low scores on the SRS may indicate high levels of social competence (Constantino & Gruber, 2012). The SRS-2 provides T-scores (M = 50, SD = 10). The Social Communication and Interaction (SCI) composite score was used in the current study. The SRS-2 has good psychometric properties (test–retest reliability ranging 0.88-0.95, internal consistency = 0.95, sensitivity = 0.92, specificity = 0.92; Constantino et al., 2012). Moderate to high correlations with other measures of social communication and behavior have also been demonstrated. The SRS has been validated in children with and without ASD. In the current study, acceptable internal consistency in the overall sample (0.74) and clinical groups (ASD = 0.70, SAs = 0.70) was demonstrated on the SCI composite.

Adaptive functioning, a collection of pragmatic abilities related to communication, social skills, and self-care, was measured using the Vineland Adaptive Behavior Scales, Second Edition parent-report form (Vineland-II; Sparrow et al., 2005). The Vineland-II yields four domain standard scores: Communication, Daily Living Skills, Socialization, and Motor Skills, and one overall composite score, the Adaptive Behavior Composite. The Socialization and Communication standard scores were used in the current study. The Socialization scale (Vineland-Socialization) consists of three subscales; Interpersonal Relationships, how an individual interacts with others, Play and Leisure Time, how the individual plays and uses leisure time, and Coping Skills, how the individual demonstrates responsibility and sensitivity to others. The Communication scale (Vineland-Communication) consists of three subscales; Receptive Language, understanding of spoken language, Expressive Language, what an individual says and how they use language to gather and provide information, and Written Skills, what an individual reads and writes. Higher scores indicate better adaptive functioning in the measured domains. The Vineland-II is a well-validated measure with strong psychometric properties (internal consistency ranges from mid 0.80 for subdomain scores to low 0.90 s for the overall composite score; test–retest is reported as 0.85 or higher, and convergent validity with the Adaptive Behavior Assessment System has been demonstrated; Sparrow et al., 2005). The Vineland-II is frequently used with clinical populations.

The Peer Social Contact Questionnaire (PSCQ) is a parent report measure previously used to measure peer relationships in children with ASD and other developmental disabilities (Guralnick, 1997; Estes et al., 2018). The PSCQ assesses the quality of relationships with up to five peer-playmates over the prior three months. Number of Peer-Playmates is defined as the number of peers (from 0 to 5) the child plays with outside of pre-arranged group activities. Each identified peer is evaluated for Conflict with Peers and Parent Support needed during play. Conflict with Peers is comprised of two questions: (1) How well does the child get along with the specified playmate (1 = *very well,* 2 = *okay,* 3 = *not very well*) and (2) How often do they have conflicts during play (1 = *frequently,* 2 = *occasionally,* 3 = *rarely;* items on this scale were reversed scored). These items were averaged across each playmate and then added to create a Conflict with Peers composite score. Higher scores indicate more conflict. Parent Support needed during play with each peer playmate in the areas of managing emotions, understanding social rules, understanding how to play activities, getting the play session started, remaining involved in play, and managing conflicts was based on 6 items. Each item was rated from 1 to 3 (1 = *frequently*, 2 = *occasionally*, and 3 = *little-to-none)*. Items for each question were reversed scored so that higher scores indicated greater need for parent support, averaged across each playmate, and then added to create a Parent Support composite score. In the current study, the overall Cronbach’s alpha for the Conflict with Peers composite was 0.70 (ASD = 0.68 and SAs = 0.71). The Cronbach’s alpha for the Parent Support composite was 0.89 for the overall sample (ASD group = 0.84 and SAs group = 0.89).

### Statistical Analysis

Analysis of covariance controlling for communication skills (Vineland-Communication) was used to evaluate group differences on measures of social functioning (SRS-2 SCI, Vineland-Socialization, Conflict with Peers, and Parent Support, Number of Peer-Playmates). Vineland-Communication was chosen as a covariate over verbal IQ because the Vineland has shown sensitivity to functional impairments among children with average (or greater) intellectual functioning (Saulnier & Klin, 2007). Therefore, the Vineland-Communication domain was chosen to parse out the effects of everyday communication impairments on social functioning. Data was visually inspected for normality and outliers using histograms and normal Q-Q plots. No outliers (> 3 SD) were detected. Non-normal distributions were found on the SRS-2 SCI in the TD group, Vineland-Communication in ASD group, Sensory Total in SPD and TD, and Conflict with Peers, Parent Support, and Number of Peer-Playmates in all groups. In addition, homogeneity of variance violations for all social functioning outcome measures (except for Vineland-Socialization) was detected, therefore, robust estimation using Bootstrapping (1000 samples) was applied for all ANCOVA analyses (Field, 2018). Assumptions regarding homogeneity of regression slopes were met.

The relationship between SAs (CSP-2) and social functioning (SRS-2 SCI, PSCQ, Vineland-Socialization), controlling for communication skills (Vineland-Communication) was examined using linear regression analysis. CSP-2 domain scores were highly correlated and added together (Sensory Total) to reduce multicollinearity (see Table 2). Data was visually examined using histograms, normal p-p plots, and scatterplots for linearity, homoscedasticity, and normality of the residuals. Robust estimation using Bootstrapping (1000 samples), was applied to regression analysis for the Parent Support model due to slight violations of normality and to the Number of Peer-Playmates model due to violations of homoscedasticity (Field, 2018). One outlier for the Vineland-Socialization, Conflict with Peers, and Parent Support Models was detected. Analyses were run with and without the outlier but the overall results did not change. Final models including the multivariate outlier are reported.

**Table 2:** Correlations for social functioning and sensory processing variables

Variable	1	2	3	4	5	6	7	8	9	10	11
1. SRS-2 SCI^a^	–										
2. Number of Peer-Playmates	0.35**										
3. Conflict with Peers	0.38**	-0.30**									
4. Parent Support	0.64**	-0.39**	0.60**								
5. Vineland-Communication	− 0.67**	0.33**	− 0.23*	− 0.36**							
6. Vineland-Socialization	− 0.79**	0.32**	− 0.33**	− 0.45**	0.74**						
7. Avoidance	0.84**	− 0.26**	0.32**	0.54**	− 0.58**	− 0.68**					
8. Registration	0.76**	− 0.17	0.25**	0.40**	− 0.53**	− 0.59**	0.78**				
9. Seeking	0.70**	− 0.11	0.28**	0.37**	− 0.50**	− 0.50**	0.75**	0.83**			
10. Sensitivity	0.83**	− 0.33**	0.35**	0.55**	− 0.60**	− 0.63**	0.89**	0.84**	0.81**		
11. Sensory Total	0.84**	− 0.23**	0.32**	0.50**	− 0.59**	− 0.65**	0.92**	0.93**	0.91**	0.95**	–

^a^Social responsiveness scale social communication and interaction

*p < 0.05

**p < 0.01

Bonferroni correction was applied to adjust for the number of comparisons performed (∝ = 0.005). All analyses were performed using SPSS version 25. Data were collected between July 2015 and March 2019.

## Results

### Description of Social Functioning in Children with SAs

**Fig. 1:**
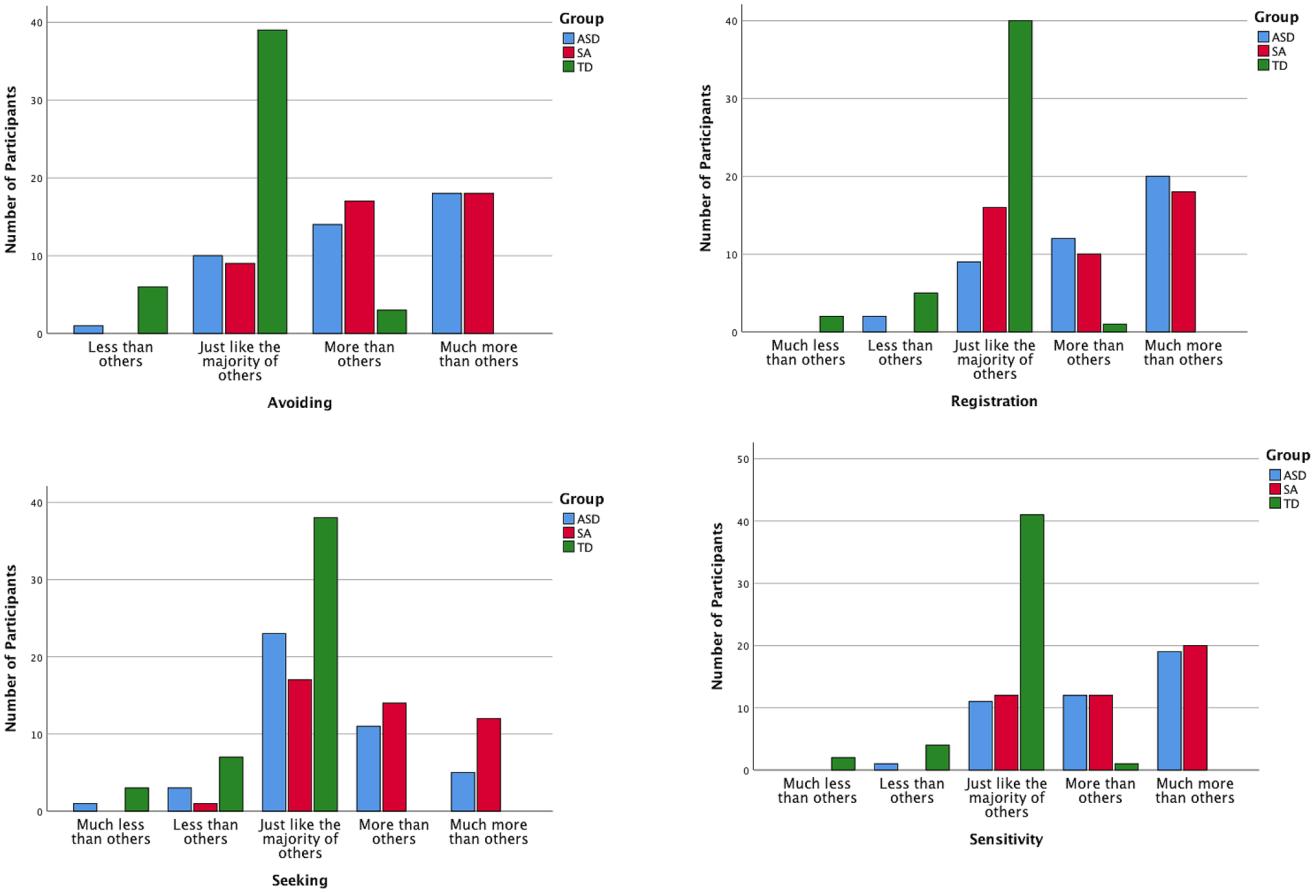
Distribution of CSP-2 scores by group

On the Vineland-Socialization, children with SAs obtained a mean standard score indicating below average social skills compared to the standardization sample. On the SRS-2 SCI, they scored in the moderate deficiencies range in reciprocal social behavior compared to the standardization sample. Because the PSCQ is not a norm-referenced measure, there are no clinical classifications. See Tables 2, 3, and Fig. 1 for details.

**Table 3:** Means, standard deviations, and ANCOVA statistics for social functioning by group

Measure	SAs			ASD			TD			ANCOVA		
	M	EMM	SD	M	EMM	SD	M	EMM	SD	*F* ratio	*df*	Partial $\eta$^2^
SRS-2 SCI^a^	67.47	66.13	11.26	71.98	69.50	10.67	44.49	47.92	5.39	44.59***	2128	0.41
Vineland-socialization	81.20	84.58	12.27	77.98	83.30	12.76	104.17	96.65	13.402	11.564***	2124	0.16
Conflict with Peers	3.11	3.09	0.77	3.01	2.98	0.78	2.60	2.64	0.52	3.12*	2117	0.05
Parent Support	8.94	8.89	2.89	9.67	9.57	2.84	6.58	6.71	0.76	10.179***	2115	0.15
Number of Peer-Playmates	3.55	3.66	1.43	2.64	2.84	1.74	3.96	3.68	1.06	3.96*	2127	0.06

*EMM* estimated marginal mean

*p < 0.05

***p < 0.001

^a^Social responsiveness scale social communication and interaction

### Group Differences in Social Functioning

There was a significant effect of group on reciprocal social behavior from the SRS-2 SCI after controlling for communication skills (Vineland-Communication) (see Table 3). *Post-hoc* analysis revealed that children with SAs had worse reciprocal social behavior than children with TD (*p* = 0.001). Children with SAs and children with ASD did not differ in their reciprocal social behavior (*p* = 0.149).

There was a significant effect of group on social skills from Vineland-Socialization after controlling for communication skills. *Post-hoc* analysis revealed that children with SAs had worse social skills than children with TD (*p* = 0.001). Children with SAs and children with ASD did not differ in their social skills (*p* = 0.599).

There was a significant effect of group on Conflict with Peers and Parent Support from the PSCQ after controlling for communication skills. *Post-hoc* analysis revealed that children with SAs had more conflict with their peer-playmates than children with TD (*p* = 0.008) but similar levels of conflict as children with ASD (*p* = 0.511). Children with SAs also needed more parent support during play with peer-playmates than children with TD (*p* = 0.002) but needed similar levels of parent support during play as children with ASD (*p* = 0.302). There was also a significant effect of group on Number of Peer-Playmates after controlling for communication skills, however, this effect did not survive correction for multiple comparisons. *Post-hoc* analysis revealed that children with SAs had a similar number of peer-playmates as children with TD (*p* = 0.930) but more peer-playmates than children with ASD (*p* = 0.025). Given that this was the only domain that children with SAs and children with ASD differed significantly, follow-up correlational analyses were performed to determine if restricted and repetitive behaviors (ADOS-2 RRB total score) were associated with number of peer-playmates, but all associations were non-significant (ASD group *r* = − 0.011, *p* = 0.943; SAs group *r* = 0.071, *p* = 0.652, TD group *r* = − 0.196, *p* = 0.183). Exploratory analysis in the two clinical groups only (SAs and ASD) was carried out to determine if the presence of ADHD symptoms explained group differences on outcome measures but the pattern of results did not differ.

### Relationship Between SAs and Social Functioning

**Fig. 2:**
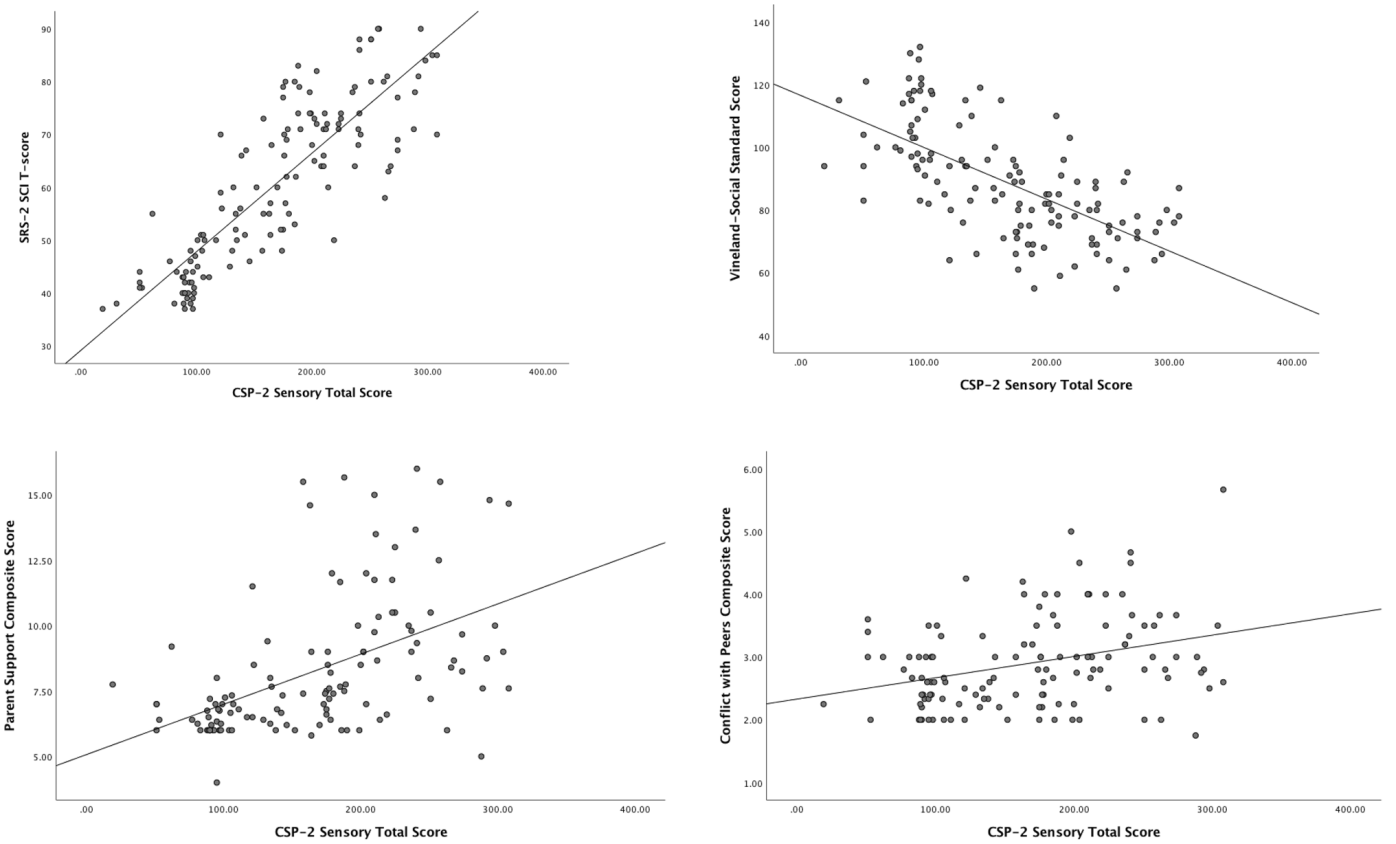
Relationship between SAs and social functioning

Separate multiple regression models were run to investigate the relationship between social functioning and SAs across the combined sample (ASD + SAs + TD) of 135 participants (Table 4). SAs (CSP-2) and communication skills (Vineland-Communication) explained significant variance in reciprocal social behavior on the SRS-2 SCI (*F*(3,128) = 219.65, *p* < 0.001, *R*^*2*^ = 0.75). Communication skills contributed 4% unique variance while SAs contributed 30% unique variance. As communication skills decreased, deficits in reciprocal social behavior increased and as SAs increased, deficits in reciprocal social behavior increased (Fig. 2).

**Table 4:** Linear regression analyses: relationship between sas and social functioning

Measure	*B*	*SE*	*t*	*p*	95% CI	*r*_a(b.c)_
SRS-2 SCI^b^						
Vineland-Communication	− 0.27	0.06	− 4.79	< 0.001	[− 0.386, − 0.160]	− 0.21
Sensory Total	0.15	0.01	12.46	< 0.001	[0.127, 0.175]	0.55
Vineland-Socialization						
Vineland-Communication	0.65	0.08	7.86	< 0.001	[0.484, 0.81]	0.44
Sensory Total	− 0.08	0.02	− 4.46	< 0.001	[− 0.113, − 0.043]	− 0.25
Conflict with Peers						
Vineland-Communication	− 0.004	0.005	− 0.68	0.50	[− 0.014, 0.007]	− 0.06
Sensory Total	0.003	0.001	2.41	0.018	[0.000, 0.005]	0.21
Parent Support^a^						
Vineland-Communication	− 0.02	0.017	− 1.03	0.281	[− 0.051, 0.015]	− 0.08
Sensory Total	0.02	0.004	4.34	0.002	[0.009, 0.026]	0.35
Number of Peer-Playmates^a^						
Vineland-Communication	0.031	0.010	2.87	0.004	[0.009, 0.049]	0.24
Sensory Total	− 0.001	0.002	− 0.45	0.642	[− 0.006, 0.003]	− 0.04

Vineland-communication is centered

^a^Confidence intervals for parameter estimates are bootstrapped

^b^Social responsiveness scale social communication and interaction

SAs and communication skills explained a significant amount of variance in social skills on the Vineland-Socialization (*F*(2,125) = 96.60, *p* < 0.001, *R*^2^ = 0.61). Communication skills contributed 19% unique variance while SAs contributed 6% unique variance. As communication skills increased, social skills increased and as SAs increased, social skills decreased (Fig. 2).

SAs and communication skills contributed significant variance in Conflict with Peers on the PSCQ (*F*(2,118) = 6.39, *p* = 0.002, *R*^2^ = 0.10). Communication skills did not contribute unique variance in Conflict with Peers. SAs contributed 4% unique variance and as SAs increased, Conflict with peers increased (Fig. 2). SAs and communication skills contributed significant variance in Parent Support on the PSCQ (*F*(2,116) = 19.35, *p* < 0.001, *R*^2^ = 0.25); however, only SAs contributed unique variance (12%) to Parent Support. As SAs increased more parent support during play with peer-playmates was needed (Fig. 2). SAs and communication skills contributed significant variance in Number of Peer-Playmates (*F*(2,128) = 7.711, *p* = 0.001, *R*^2^ = 0.11). Communication skills contributed 6% unique variance in Number of Peer-Playmates and as communication skills increased, Number of Peer-Playmates increased. SAs did not contribute unique variance in Number of Peer-Playmates. Exploratory analysis in the two clinical groups only (SAs and ASD) was carried out to determine if the presence of ADHD symptoms explained significant variance in the outcome. ADHD symptoms did not explain significant variance for any of the social functioning outcomes except for Parent Support. There was a marginally significant group by ADHD symptom interaction (*F*(1,68) = 4.001, *p* = 0.049). Parent Support was greater for children with ASD and ADHD symptoms than for children with SAs and ADHD symptoms. Parent Support did not differ for children with no ADHD symptoms. Sensory abnormalities explained significant variance in Parent Support for both groups.

## Discussion

This study investigated social functioning in school-age children with SAs, and a clinical comparison group of children with ASD and children with TD. Children with SAs demonstrated impairments in social functioning on standardized measures of reciprocal social behavior and social skills, with means scores on these measures falling within the moderate impairment range. When group differences were investigated, controlling for communication skills, children with SAs demonstrated worse social functioning than children with TD. Interestingly, their scores on standardized measures of social functioning were similar to children with ASD. These findings are consistent with prior research demonstrating lower than expected social skills in school-age children with SAs (Ben-Sasson et al., 2009; Armstrong et al., 2013).

Group comparison, controlling for communication skills, also revealed that children with SAs experienced more conflict with their peer-playmates than children with TD. Children with SAs and children with ASD experienced similar levels of conflict during play with peer-playmates. Although level of conflict with peer-playmates was assessed through parent report, this finding converges with existing evidence using observations of peer interactions (Cosbey et al., 2012). Higher levels of conflict are associated with fewer interactions with peers in children with SAs (Cosbey et al., 2012), children with TD (Gottman, 1983; Ladd, 1992), and children with ASD (Frankel & Mintz, 2011) and is associated with peer rejection (McElwain et al., 2010). This could mean that children with SAs have fewer opportunities to develop their social skills due to reduced peer interactions and may experience more rejection than their socially skilled peers. High rates of co-occurring psychological symptoms in the SAs group may also have contributed to difficulties with peer interactions and, more generally, with social functioning. Future research will be needed to explore this idea further.

We found that children with SAs required more parent support during play interactions with peer-playmates than children with TD and a similar level of support as children with ASD. Parental support activities involved support with managing conflict and emotions, understanding social rules, and providing help with foundational play skills. To our knowledge, this is the first study reporting on parent support activities during play with peer-playmates in children with SAs. These findings are consistent with prior research in which parent support was increased for children with ASD (Estes et al., 2018). Direct parent involvement during peer interactions may be a sign of poorer social skills (Mikami et al., 2010). In the current study, increased parent support was associated with decreased social skills and increased impairments in reciprocal social behavior, providing some initial support for this theory.

Trend-level differences in Number of Peer-Playmates was found. Number of peer-playmates, as measured by parent report, were similar in children with SAs and children with TD and both groups had a greater Number of Peer-Playmates than children with ASD. Although prior research has suggested that children with ASD have fewer peer-relationships than children with TD (Petrina et al., 2014), it is surprising that children with SAs had more peer-playmates than children with ASD, especially in light of similarities in social functioning. Although there is significant heterogeneity in ASD, differences between children with SAs and ASD in number of peer-playmates could be due to differences in motivation to seek out peer-playmates (Calder et al., 2013; Sedgewick et al., 2016). Children with SAs, despite having social challenges, were perhaps more motivated to spend time with peers engaged in play dates. Differences in restricted and repetitive behaviors (RRB) may also account for differences in number of peer-playmates (Jones et al., 2017). For example, children with ASD may be more focused on discussing special interests than on socializing when interacting with peers or may have difficulties developing relationships with peers due to behavioral rigidities (e.g., needing to direct the play of others). To explore this possibility in the current sample, correlations between RRBs and number of peer-playmates were calculated for each group separately, but no significant relationships were found. However, no research, to our knowledge, has reported on the number of peer-playmates in children with SAs and possible explanations about differences across groups need to be explored further.

Similarities in social functioning and presence of sensory abnormalities in the SAs and ASD groups might suggest that children with SAs lie somewhere along the autism spectrum or have characteristics of the broader autism phenotype (BAP). To date, studies on BAP traits have primarily focused on relatives of children with ASD and adult populations (Ozonoff et al., 2014; Landry & Chouinard, 2016; Rubenstein & Chawla, 2018). Further research is needed to determine whether BAP traits might extend to children without a family history of ASD such as the SAs group in this study. Further, it is notable that in the SAs group the overall calibrated severity score on the ADOS-2, an index of autism-related symptoms, fell in the low range. Suggesting that, unlike children with ASD, social functioning in children with SAs is not impaired in the context of a one-on-one interaction with an examiner. Future research is needed to understand the impact of different contexts on social functioning and, more generally, the concordant and discordant features of children with SAs and ASD.

The third aim of the study was to determine the relationship between SAs and social functioning by examining SAs on a continuum across the entire sample of children. We found that increased SAs were associated with worse reciprocal social behavior and social skills, explaining a moderate to small proportion of unique variance (33% and 6% respectively) after accounting for communication skills. The current findings are congruent with prior research in children with ASD where moderate to strong relationships between SAs and reciprocal social behavior (Hilton et al., 2007, 2010) and social skills (Liss et al., 2006) were found. It is notable that the strongest relationships were apparent between SAs and reciprocal social behavior (as measured by the SRS) compared with social skills (as measured by the Vineland) across these studies, a pattern also reflected in our own data. This could reflect measurement overlap between the CSP-2 and SRS-2 (see limitations section for further discussion) or clarify the specific components of social functioning that are most affected by SAs. Our findings also extend prior research by examining associations between SAs and social functioning across a broader sample of children than has been previously investigated (i.e., including children with SAs).

Finally, we found that SAs were associated with increased peer conflict and parent support after accounting for communication skills but were not related to Number of Peer-Playmates. Previous research, to our knowledge, has not reported on the relationship between SAs and these specific aspects of social functioning in children with and without TD. One interpretation of our results is that SAs prevent children from being fully engaged in social interactions (Doble & Magill-Evans, 1992). They may be distracted by SAs or may spend their cognitive resources compensating for sensory processing disruptions, the consequence of which may be fewer resources for recognizing social cues, managing their own emotions, and adequately managing conflict as it arises.

Social skills interventions targeting both broader (e.g., reading social cues) and specific social skills (e.g., how to respond when a conflict during play arises) may be beneficial for children with SAs. Although there is little empirical evidence on social skills interventions in children with SAs without ASD, research on social skills interventions in children with ASD could be considered to support children with SAs. Peer-mediated and school-based interventions have resulted in improved social skills and peer interactions among school-aged children with ASD (Kasari et al., 2012; Locke et al., 2019). A school-based intervention program that combines individualized social skills instruction, in vivo practice of learned social skills, and peer support may be particularly beneficial (Locke et al., 2019). Importantly, there is evidence suggesting that the presence of co-occurring psychiatric symptoms could affect social skills interventions. For example, Antshel et al. (2011) found that children with ASD and children with ASD and co-occurring anxiety showed improved social skills after a 10-week social skills intervention focused on conversational skills and social problem solving but that children with ASD and co-occurring ADHD did not. This study demonstrated that children with SAs have higher rates of co-occurring psychiatric symptoms, in particular ADHD. Thus, designing and implementing interventions that address co-occurring psychiatric disorders may be critical to improve social skills for children with SAs.

### Limitations

Several limitations in the current study should be noted. Small numbers of non-white and female participants limits generalizability of the results. Our findings may be most relevant for verbally fluent children with Average to High Average intellectual functioning since this sample did not include children with intellectual disability. Research including children across the entire spectrum of intellectual functioning is needed to provide further insight into the nature of the relationship between sensory abnormalities and social functioning. Further, it is possible that the use of the WASI instead of the WASI-II could have resulted in a slight over-estimate of IQ scores, which would have been consistent across all groups in the study (Trahan et al., 2018). Additional, clinical characterization of children with SAs with respect to intervention history, school supports, SES, and family environment will also be important in future studies.

The measure of peer interaction, the PSCQ, limits parents report to a maximum of five peer-playmates. This allows parents to provide greater detail about each peer-playmate. However, it limits the PSCQ as a quantitative measure as it may not capture the full variability in number of peer-playmates. Future research could better address this question by adding a measure of the total number of peer-playmates for each child. It should also be noted that the SRS-2 probes sensory experiences, however, given the small number of items (< 3 of 65), these did not likely influence the results. Finally, measurement of sensory symptoms relied solely on parent-report. There may be differences between direct observation of SAs using laboratory-based tests versus parent report (Schoen et al., 2009). However, parent-report provides an ecologically valid assessment of SAs. Thus, future research should consider including both. Cross-informant ratings may offer further insight into social functioning across different social contexts. For example, there is some suggestion that in children with ASD teachers may report better skills in responding to and maintaining social interactions than parents (Murray et al., 2009).

In our sample, there were high rates of co-occurring psychiatric symptoms in the SAs group. Future research should investigate the relationship between co-occurring psychiatric symptoms and social functioning in children with SAs given evidence that social functioning problems are associated with ADHD (Maedgen & Carlson, 2000), anxiety (Riby et al., 2014), and depression (Verboom et al., 2014). Although it was outside of the scope of the current paper to investigate of the relationship between all co-occurring psychiatric symptoms and social functioning, future research is needed to evaluate the relationship of social functioning to other types of psychiatric symptoms beyond those evaluated in this study.

## Conclusion

This study provides some of the first empirical evidence that children with SAs have worse social functioning than children with TD but similar social functioning to children with ASD. This has several implications for clinical practice. This study’s findings suggest that social skills interventions should be considered for children with SAs, even those who do not meet criteria for a developmental disability or psychiatric diagnosis. Further studies are needed to determine whether children with SAs may benefit from social skills interventions that increase social skills and are effective for children with co-occurring psychiatric diagnoses.
